# Pushing to the Limits: What Processes during Cognitive Control are Enhanced by Reaction–Time Feedback?

**DOI:** 10.1093/texcom/tgab027

**Published:** 2021-04-07

**Authors:** Astrid Prochnow, Moritz Mückschel, Christian Beste

**Affiliations:** Cognitive Neurophysiology, Department of Child and Adolescent Psychiatry, Faculty of Medicine, Technische Universität Dresden, D-01309 Dresden, Germany; Cognitive Neurophysiology, Department of Child and Adolescent Psychiatry, Faculty of Medicine, Technische Universität Dresden, D-01309 Dresden, Germany; Cognitive Neurophysiology, Department of Child and Adolescent Psychiatry, Faculty of Medicine, Technische Universität Dresden, D-01309 Dresden, Germany

**Keywords:** conflict, EEG signal decomposition, flanker, selective attention

## Abstract

To respond as quickly as possible in a given task is a widely used instruction in cognitive neuroscience; however, the neural processes modulated by this common experimental procedure remain largely elusive. We investigated the underlying neurophysiological processes combining electroencephalography (EEG) signal decomposition (residue iteration decomposition, RIDE) and source localization. We show that trial-based response speed instructions enhance behavioral performance in conflicting trials, but slightly impair performance in nonconflicting trials. The modulation seen in conflicting trials was found at several coding levels in EEG data using RIDE. In the S-cluster N2 time window, this modulation was associated with modulated activation in the posterior cingulate cortex and the superior frontal gyrus. Furthermore, in the C-cluster P3 time window, this modulation was associated with modulated activation in the middle frontal gyrus. Interestingly, in the R-cluster P3 time window, this modulation was strongest according to statistical effect sizes, associated with modulated activity in the primary motor cortex. Reaction–time feedback mainly modulates response motor execution processes, whereas attentional and response selection processes are less affected. The study underlines the importance of being aware of how experimental instructions influence the behavior and neurophysiological processes.

## Introduction

Human goal-directed behavior depends on a multitude of cognitive processes, including selective attention and response selection processes. Selective attention is often referred to in a “spotlight” metaphor (Posner 1980; Goodhew et al. 2016), and therefore can be either spatially broadly or narrowly distributed (Wachtel 1967; Hübner et al. 2010). The effect of the width of attentional focus can be demonstrated, for example, by the Eriksen flanker paradigm (Eriksen BA and Eriksen CW 1974; Hübner et al. 2010). In congruent trials of a flanker paradigm, spatially broadly distributed attention might be beneficial, because the flankers share the information of the target and therefore facilitate the choice of the correct response. In incongruent trials, on the other hand, spatially narrowly distributed attention might be beneficial, because the flankers carry conflicting information and thereby impede the selection of the correct choice (Hübner et al. 2010). The width of the attentional focus and, therefore, the attention to relevant stimuli rather than distractors can be modulated by top-down processes (Reynolds and Chelazzi 2004; Gazzaley and Nobre 2012). These top-down processes can be modulated by the task instructions at the beginning of a task (Zanto and Gazzaley 2009; Rutman et al. 2010), but also by refreshing task instructions during the task (Camos et al. 2018).

A widely used instruction in psychological experiments—besides task-specific instructions—is to respond as accurately and as quickly as possible. Some experiments focus on one part of the instruction to induce different strategies, so either accuracy or speed is more emphasized (Standage et al. 2014). In the case of the focus on speed, it is possible to present the instruction in a trial-based manner (Franklin and Okada 1983; Touron and Hertzog 2014), for example, giving a hint when reaction times (RTs) exceed a set time limit. Trial-based feedback to response speed is an integral part of experimental approaches in areas of cognitive neuroscience dealing with the evaluation of conflict monitoring, response inhibition, and error processing. However, even though such instructions belong to the standard repertoire of experimental manipulations in cognitive neuroscience and psychology, the processes induced by such a feedback and their neurophysiological correlates are less clear. To which extent are certain aspects of information processing modulated by trial-based feedback of the RT?

On the one hand, time pressure may have negative effects on performance by decreasing the time used for stimulus encoding and reducing the quality of sensory filtering (Dambacher and Hübner 2015). On the other hand, effects of such reaction–time feedback might purely speedup processing (Franklin and Okada 1983; Touron and Hertzog 2014). Yet, it is also possible that reaction–time feedback is a more general “refreshing cue” affecting processes besides speed, thereby sharpening the focus of attention to relevant stimuli (Camos et al. 2018) and speeding up cognitive processes (Standage et al. 2014). In a flanker paradigm, feedback purely based on response speed should facilitate responses in both conditions. However, if the effects of reaction–time feedback mostly affect the cognitive level, the effects of reaction–time feedback should differ between conditions. A narrowing of the attentional focus should improve performance in the incongruent condition, as the distracting flanker information will be processed less, but impair performance in the congruent condition as the supporting flanker information will be processed less. A broadening of attentional focus should have the opposite effects on the conditions (Hübner et al. 2010). In general, stress or time pressure can cause a broader attentional focus with higher distractibility and worse quality of sensory filtering (Sänger et al. 2014; Dambacher and Hübner 2015; Qi et al. 2018). Considering these previous findings and assumptions, in the current study, the processes within the trial immediately after trial-based feedback are of interest, where reaction–time feedback might indeed serve as a refreshing cue (Camos et al. 2018), and therefore cause a narrowing of attentional focus in the following trial.

We use electroencephalography (EEG) data to examine the modulations of certain aspects of information processing by trial-based reaction–time feedback. However, in classical event-related potentials (ERPs), aspects associated with stimulus processing, response selection, and motor execution are intermingled (Mückschel et al. 2017a; Chmielewski et al. 2018). This is not only because processes occurring simultaneously overlap in EEG data (Ouyang et al. 2011), but also because EEG signals reflect a mixture of activity from different functional neuroanatomical sources (Nunez et al. 1997; Huster et al. 2015; Stock et al. 2017). This is critical for the current study because trial-based feedback may affect multiple processes from stimulus evaluation to simple motor responding. To isolate possibly differentially modulated coding levels in the neurophysiological signal, we applied residue iteration decomposition (RIDE; Ouyang et al. 2015a). RIDE is a temporal decomposition method that decomposes the EEG signal into different clusters of components, with variable intercomponent delays. For the estimation of one of the clusters, all other clusters are subtracted on the single trial level and residuals of all trials are aligned to one time point. The different clusters can be associated with different stages of cognitive processing (Ouyang et al. 2015a). Stimulus-related processes such as perception and attention can be referred to by the S-cluster, whereas the R-cluster refers to response-related processes such as response execution, and the C-cluster refers to intermediate processes such as response selection (Ouyang et al. 2011, 2017). The waveforms returned by the RIDE algorithm can be interpreted similarly to classical ERPs, but with the advantage that the underlying processes can now be clearly attributed to specific cognitive processing steps.

For the S-cluster, the narrowing of the attentional focus and the inhibition of distracting stimuli (Diamond 2013; Moher et al. 2014) might be associated with modulations in the P1 time window. The P1 reflects the inhibition of task-irrelevant information and the enhancement of processing of relevant information (Klimesch 2011; Wolff et al. 2018). In the N2 time window, perceptual processes reflecting a mismatch detection on the one hand, and response-related processes such as conflict processing on the other hand (Folstein and Van Petten 2008) can be disentangled by RIDE (Mückschel et al. 2017a; Chmielewski et al. 2018). In the analyses of the effects of reaction–time feedback, this separation might lead to distinguishable elements of the N2 component, which can be associated with early modulations of the perceptual inhibition of the flanker stimuli according to the narrowing of attentional focus (Diamond 2013; Moher et al. 2014) as well as later monitoring of the conflicting information (Folstein and Van Petten 2008). The stimulus-related aspects of processes in the N2 time window (Friedrich and Beste 2018) may be modulated by selective attention and reflect the inhibition of processing irrelevant stimuli (Wascher et al. 2012; Sänger et al. 2014; Qi et al. 2018). Such processes are thought to be associated with networks involving the frontal and parietal brain areas (Corbetta and Shulman 2002).

Furthermore, a mixture of different processes is not only evident in the N2 time window, but may also be a relevant issue for the P3 time window (Ouyang et al. 2011, 2017; Mückschel et al. 2017a). On the one hand, the P3 component has been associated with stimulus–response mapping and response selection processes (Verleger et al. 2005; Twomey et al. 2015), which are reflected by the RIDE C-cluster (Ouyang et al. 2011, 2017). On the other hand, remapping of stimulus–response associations especially in conflicting conditions and motor response preparation processes are evident (Mückschel et al. 2017a; Brydges et al. 2020), which are reflected by the RIDE R-cluster (Ouyang et al. 2011, 2017). As previous studies found conflict processing and reaction–time feedback to affect response selection and purely motor-associated processes (Rinkenauer et al. 2004; Bluschke et al. 2017), modulations by conflict and reaction–time feedback should be evident at both coding levels. For the response selection processes, these modulations might depend on the left dorsolateral prefrontal cortex (dlPFC), as the dlPFC is especially involved in response selection processes when conflicting responses are evident, attention is required, and task conditions are demanding (Hadland et al. 2001; Vallesi et al. 2011). Especially, the left dlPFC is associated with the adaptation of strategies in terms of the speed–accuracy trade-off (Vallesi et al. 2012). As the R-cluster refers to response preparation, including remapping of stimulus–response associations and execution, modulations at this level should be related to premotor and motor areas (Cheney 1985).

## Materials and Methods

### Sample

We report how we determined our sample size, all data exclusions (if any), all inclusion/exclusion criteria, whether inclusion/exclusion criteria were established prior to data analysis, all manipulations, and all measures in the study. A group of *N* = 37 healthy adults (age: 24 ± 3 years, 22 males) participated in the study. None of them reported physical, psychiatric, or neurological illness in a brief telephone interview. One participant was excluded because of scores above cutoff (*T*-values ≥70) in the “Adult Self-Report for Ages 18–59” (Achenbach 2015), and 9 further participants were excluded due to an insufficient number of postspeedup trials for statistical analyses in the behavioral data (≤5 trials). In the end, the data of *N* = 27 subjects were analyzed (age: 24 ± 3 years, 15 males). All participants provided written informed consent before any study procedure was applied. The study was approved by the local ethics committee of the Medical Faculty of the Technische Universität Dresden.

To determine the detectable effect size, a sensitivity analysis was conducted using G*Power (Faul et al. 2007). With the total sample size of *N* = 27 subjects and using repeated-measures analysis of variance (ANOVA) with a 2 × 2 design, medium effect sizes (η^2^ = 0.077) can be detected with a power of 95%. No part of the study was preregistered. No analyses were preregistered.

### Task

**Figure 1:**
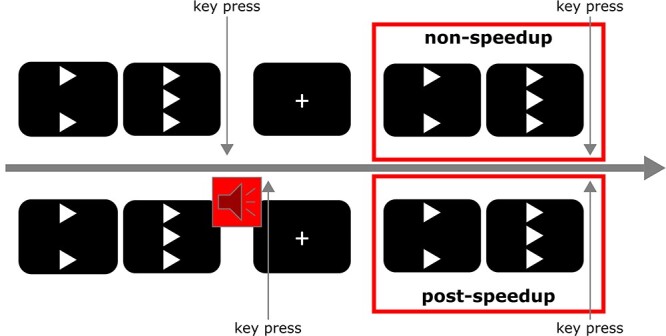
Task procedure for 2 successive trials. The typical procedure of a nonspeedup trial is displayed in the upper panel; and the typical procedure of a postspeedup trial is displayed in the lower panel. For reasons of simplicity, the figure only contains congruent trials. Markings on the timeline are not to scale.

Participants were seated in front of a 24-inch thin-film-transistor (TFT) monitor with a refresh rate of 144 Hz at a distance of about 60 cm. To present the stimuli and for recording of the behavioral data, “Presentation” software (Version 17.1, Neurobehavioral Systems, Inc., www.neurobs.com) was used. Before the experiment was started, participants completed 20 practice trials supervised by an examiner after oral and written instruction. They were told to use the left and right “Ctrl” keys to respond to the stimuli and to respond as fast and as accurately as they could. At the beginning of each trial, 2 white vertically aligned arrowheads (flankers) were shown one above the other with a space for the target stimulus in between. After 200 ms, another white arrowhead (target) was displayed between the 2 flankers. This central arrowhead pointed either in the same direction as the flanker arrowheads (congruent condition) or in the opposite direction (incongruent condition). The participants were asked to indicate the direction of the target arrowhead by keypress (i.e., left Ctrl key for leftwards and right Ctrl key for rightwards, respectively) with their right and left index fingers. Congruent and incongruent trials occurred pseudorandomly in a ratio of 2:1, respectively. The flankers and target were shown on the screen for another 300 ms. Afterwards, until the end of the trial, the screen turned black. To put time pressure on the participants and thus be able to study the effects of reaction–time feedback on cognitive processes, a short warning tone was presented 350 ms after the target onset via headphones as negative reaction–time feedback if there had been no reaction by then (i.e., “speedup” signal). The intertrial interval (ITI) started either after a response was given or after the speedup signal was presented. Responses, even if given after a speedup signal, were classified as valid if RTs were between 100 and 1000 ms. The ITI varied randomly between 900 and 1300 ms. During the ITI, a white fixation cross was displayed in the middle of the screen until the next trial started. Analyses were conducted for trial *n* dependent on whether a speedup signal occurred in trial *n*−1 (postspeedup vs. nonspeedup). The task procedure for 2 successive trials is illustrated in Figure 1. For the analyses, accuracy and RTs were used separated in congruent versus incongruent as well as in postspeedup versus nonspeedup trials.

### E‌EG Recording and Analysis

The EEG was recorded from 60 equidistant passive Ag/AgCl electrodes with a sampling rate of 500 Hz using a BrainAmp direct current (DC) amplifier (BrainProducts, Inc.). Impedances were kept under 5 kΩ. The reference electrode was placed at position FPz, the ground electrode was placed at θ = 58 and φ = 78. Preprocessing of the data was done offline using the BrainVision Analyzer 2.1 software package (BrainProducts, Inc.). In the first step, data were downsampled to 256 Hz. An infinite impulse response (IIR) filter (zero-phase shift Butterworth filter, 0.5–40 Hz, slope 48 db/oct) was applied and technical artifacts, such as time windows with a DC-correction, were cut out from the continuous EEG signal during a raw data inspection. Periodic artifacts, such as pulse artifacts, horizontal and vertical eye movements were removed during an independent component analysis (infomax algorithm). Afterwards, data sets were segmented locked to the presentation of the flanker stimuli, with a segment size of −2000 to 2000 ms. For ERP analysis, a second IIR filter (zero-phase shift Butterworth filter, high cutoff 20 Hz, slope 48 db/oct) was applied. Automatic artifact rejection procedure was applied to exclude remaining trials containing artifacts (amplitude criterion: 150 μV/−150 μV; maximal value difference: 150 μV in a 200 ms interval; low activity: below 0.5 μV in a 100 ms interval). Afterwards, a current source density (CSD) transformation was used to allow a reference-free evaluation of the EEG data (Nunez and Pilgreen 1991). Finally, baseline correction was applied using a baseline from −200 to 0 ms.

### RIDE

RIDE was applied to the baseline-corrected EEG data. RIDE decomposes ERPs on a single-trial level in an iterative way into static latency and variable latency components based on their timing and timing variability using robust algorithms (Ouyang et al. 2015a). Application of CSDs as a spatial filter (Nunez and Pilgreen 1991) is not critical, because RIDE does not separate component clusters by their scalp distributions and waveforms, but only by their latency variability (Ouyang et al. 2015a). To perform RIDE analysis, first, an initial estimate of the time markers of the intermediate C-cluster (“C_L_”) is made, for example, using Woody’s method (Woody 1967). Based on this, the clusters are separated from each other, as well as using the results of this separation to re-estimate the C-cluster. The S-cluster and R-cluster are derived by time markers (“latencies,” “S_L_,” and “R_L_”), that is, the time points of the respective stimulus and response onsets. To estimate S, RIDE subtracts C and R from each single trial and aligns the residual of all trials to the latency S_L_ to obtain S as the median waveform for all time points. The same procedure is used to obtain C and R. For the R-cluster, the response needs to be part of the epoch, and around 98% of all responses were carried out within this epoch. This procedure is carried out iteratively, until the deviation of the residuals from the median of all trials reaches a minimum according to the L1 norm. The RIDE decomposition was conducted according to established procedures (Ouyang et al. 2011; Verleger et al. 2014) separately for each single electrode channel (Ouyang et al. 2015b) using the RIDE toolbox (manual available on http://cns.hkbu.edu.hk/RIDE.htm). To extract the waveform of each RIDE component, a time window function is used. For the current study, this was from 200 ms prior to the flanker stimuli to 800 ms after the flanker stimuli for the S-cluster, from 100 to 1000 ms after the flanker stimuli for the C-cluster, and ±300 ms around the response trigger for the R-cluster (Ouyang et al. 2015b). After running this algorithm, RIDE returns the waveforms on each channel and topographies for each subject for each time point separate for the 3 clusters (Ouyang et al. 2015a, 2015b).

According to previous papers by our group (Bluschke et al. 2017; Mückschel et al. 2017a), the RIDE clusters can be used to quantify components corresponding to classic ERPs elicited by a flanker task. The choice of appropriate time windows and topographic locations for the RIDE components was done by visual inspection and in accordance with previous literature (Folstein and Van Petten 2008; Verleger et al. 2014; Twomey et al. 2015; Bluschke et al. 2017; Mückschel et al. 2017a). In the S-cluster, we quantified the RIDE-P1_Flanker_ (95–115 ms), the RIDE-P1_Target_ (310–330 ms), and the RIDE-N1_Target_ (385–405 ms) at electrodes P7, P8, P9, and P10, as well as the RIDE-N1_Flanker_ (153–173 ms) at electrodes P7 and P8. The RIDE-N2 in the S-cluster was divided into 2 separate time windows after visual inspection, so we quantified the RIDE-N2_first peak_ at electrode Cz (320–330 ms) and the RIDE-N2_second peak_ at electrodes Cz and FCz (455–485 ms). In the C-cluster, the RIDE-P3 (P3_C_) was quantified using a semiautomatic peak detection procedure in a time window from 450 to 700 ms at electrodes CP1, CP2, C3, C4, CP3, and CP4. For the R-cluster, the topographic location was inconclusive according to the topographic plots, so that difference waves between the postspeedup and nonspeedup condition were calculated for congruent and incongruent condition separately (please refer to Supplementary Fig. S1). Taking visual inspection of the difference topographic plots into account, we divided the R-cluster into 2 separate time windows at FCz, FC2, F2, and FC4 and quantified both peaks using a semiautomatic peak detection method. According to previous literature, this differential activation might resemble a conflict slow potential (conflict SP; West et al. 2005; Chen et al. 2011). For the first peak of the R-cluster activation (R_first peak_), we chose a time window from 380 to 540 ms, and for the second peak of the R-cluster activation (R_second peak_), we chose a time window from 560 to 700 ms.

### Source Localization

For significant interactions of reaction–time feedback and conflict in the RIDE data, source localization analyses were performed for the corresponding time windows using standardized low-resolution brain electromagnetic tomography (sLORETA; Pascual-Marqui 2002) based on the estimated RIDE clusters. The sLORETA software divides the intracerebral volume into 6239 voxels at 5 mm spatial resolution. On the basis of the MNI152 template (Mazziotta et al. 2001), the standardized current density at each voxel is computed in a realistic head model (Fuchs et al. 2002). sLORETA provides a unique solution to the inverse problem (Pascual-Marqui 2002; Marco-Pallarés et al. 2005; Sekihara et al. 2005). Reliable results without a localization bias (Sekihara et al. 2005) and the estimated sources were validated in EEG/functional magnetic resonance imaging and neuronavigated EEG/transcranial magnetic stimulation (TMS) studies (Sekihara et al. 2005; Dippel and Beste 2015). The voxel-based sLORETA images were compared between experimental conditions using sLORETA-built-in voxel-wise randomization tests with 3000 permutations, based on statistical nonparametric mapping. Voxels with significant differences (*P* < 0.050, corrected for multiple comparisons) between contrasted conditions were located in the Montreal Neurological Institute (MNI)-brain.

### Statistical Analysis

We used repeated-measures ANOVAs for the behavioral data as well as for the neurophysiological data. For the behavioral data, the accuracy as well as RTs were analyzed. Because it complicates the interpretation of the data to analyze both measurements separately (due to a speed–accuracy trade-off), we also calculated the inverse efficiency score (IES; Bruyer and Brysbaert 2011), that is, RTs were divided by accuracy as decimal number. A higher IES indicates worse performance. For the analyses, we used the within-subject factors “Congruency” (congruent vs. incongruent) and “Post-Speedup” (postspeedup vs. nonspeedup). For the neurophysiological data, the within-subject factor “Electrode” was additionally included whenever necessary. One-way ANOVAs and *t*-tests were used for post hoc analyses. For the flanker congruency effect (Eriksen BA and Eriksen CW 1974), the data in incongruent trials was subtracted from the data in congruent trials. To examine the effects of the “speedup” signal, the “speedup-effect” was calculated by subtracting the data in nonspeedup trials from the data in postspeedup trials. Bonferroni and Greenhouse–Geisser corrections were applied when necessary. To confirm the results of the interactions of “Congruency*Post-Speedup,” as this interaction refers to our main research question, we conducted Bayesian analyses as suggested by Wagenmakers (2007) using the template provided by Masson (2011). This analysis can determine the probability of the null hypothesis being true, given the observed data, *p*(*H*_0_|*D*). If this probability is <0.5, this indicates that the alternative hypothesis (i.e., an interaction exists) is more likely to be true than the null hypothesis (i.e., no interaction exists). Weak evidence is indicated by values between 0.5 and 0.75, positive evidence is reflected by values between 0.75 and 0.95, and strong evidence for the null hypotheses being true is indicated by values between 0.95 and 0.99 (Raftery 1995).

## Results

### Behavioral Data

**Figure 2:**
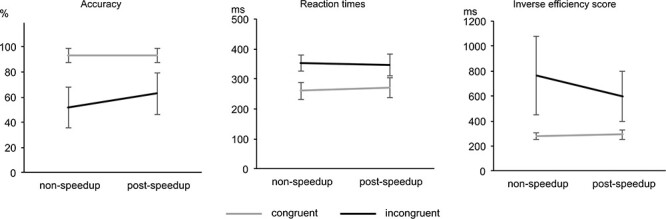
Behavioral data. Separate graphs for accuracy (left), reaction times (at center), and inverse efficiency score (right). Separate lines for the congruent (gray) and incongruent (black) condition.

For the analyses, only correct trials were used. For the congruent condition, 235 ± 34 nonspeedup trials and 62 ± 33 postspeedup trials were included in the analyses. For the incongruent condition, 65 ± 19 nonspeedup trials and 20 ± 15 postspeedup trials were included in the analyses. We did not set a cutoff for accuracy, since an outlier analyses did not reveal any outliers. The descriptive statistics of the behavioral data, that is, the accuracy, RTs and IES as a combined measure of performance for the congruent and incongruent as well as for the postspeedup and nonspeedup trials are shown in Figure 2.

A repeated-measures ANOVA of the accuracy revealed significant main effects of the factors Congruency (*F*_1,26_ = 246.55; *P* < 0.001; η*_p_*^2^ = 0.905) and Post-Speedup (*F*_1,26_ = 9.10; *P* = 0.006; η*_p_*^2^ = 0.259). Accuracy was higher for congruent (93 ± 5%) than for incongruent (57 ± 14%) trials. Also, accuracy was higher for postspeedup (78 ± 10%) than for nonspeedup (72 ± 10%) trials. Furthermore, analysis revealed a significant interaction of Congruency*Post-Speedup (*F*_1,26_ = 13.63; *P* = 0.001; η*_p_*^2^ = 0.344). The speedup-effect (i.e., the difference of postspeedup minus nonspeedup trials) was evident in the incongruent condition (*t*(26) = 3.37; *P* = 0.002) as an improvement (11 ± 17%), but not in the congruent condition (*t*(26) = 0.24; *P* = 0.812).

For the RTs, a repeated-measures ANOVA revealed a significant main effect of the factor Congruency (*F*_1,26_ = 364.17; *P* < 0.001; η*_p_*^2^ = 0.933), with larger RTs in the incongruent (350 ± 30 ms) than in the congruent (266 ± 29 ms) condition. Furthermore, analysis revealed a significant interaction of Congruency*Post-Speedup (*F*_1,26_ = 8.59; *P* = 0.007; η*_p_*^2^ = 0.248). The speedup-effect was evident in the congruent condition (*t*(26) = 3.04; *P* = 0.005) as a worsening (11 ± 19 ms), but could not be observed in the incongruent condition (*t*(26) = −1.23; *P* = 0.217).

We analyzed IES as a combined measurement, since interactions in accuracy and RTs show different patterns. The speedup-effect in accuracy is evident in the incongruent condition only, whereas the speedup-effect in RTs is evident only in the congruent condition. A repeated-measures ANOVA of the IES revealed significant main effects of the factors Congruency (*F*_1,26_ = 77.44; *P* ≤ 0.001; η*_p_*^2^ = 0.749) and Post-Speedup (*F*_1,26_ = 8.19; *P* = 0.008; η*_p_*^2^ = 0.240). IES was higher for incongruent (683 ± 227 ms) than for congruent (287 ± 30 ms) trials. Also, IES was higher for nonspeedup (524 ± 156 ms) than for postspeedup (447 ± 102 ms) trials. Furthermore, analysis revealed a significant interaction of Congruency*Post-Speedup (*F*_1,26_ = 11.32; *P* = 0.002; η*_p_*^2^ = 0.303). The speedup-effects (i.e., the difference of postspeedup minus nonspeedup trials) in the congruent and the incongruent condition differed significantly from each other (*t*(26) = 3.37; *P* = 0.002), with a large improvement in incongruent trials (−165 ± 275 ms) and a smaller but also significant worsening in congruent trials (12 ± 21 ms).

We conducted Bayesian statistics for the Congruency*Post-Speedup interactions concerning all 3 behavioral measures to validate our findings. Values of *p*(*H*_0_|*D*) < 0.001 were obtained for all of them, indicating that the alternative hypothesis is likely to be true for all 3 measures. Furthermore, we compared the behavioral data in nonspeedup and postspeedup trials with a drift diffusion model approach in terms of the dual-stage two-processes (DSTP) model as described by Hübner et al. (2010), using the R-package “flankr” provided by Grange (2016). We found a larger drift rate of the target in postspeedup trials (0.069 ± 0.066) compared with nonspeedup trials (0.038 ± 0.035; *t*(26) = −2.24, *P* = 0.034), whereas the drift rate of the late stage of stimulus selection was larger in nonspeedup (0.800 ± 0.148) than in postspeedup trials (0.716 ± 0.170; *t*(26) = 2.16, *P* = 0.040). A more detailed description of the procedures and results can be found in the Supplementary Material S1.

### Neurophysiological Data: RIDE ERPs

For the neurophysiological data analyses, only correct trials were used. For the congruent condition, 153 ± 35 nonspeedup trials and 39 ± 23 postspeedup trials were included in the analyses. For the incongruent condition, 42 ± 16 nonspeedup trials and 14 ± 12 postspeedup trials were included in the analyses.

**Figure 3:**
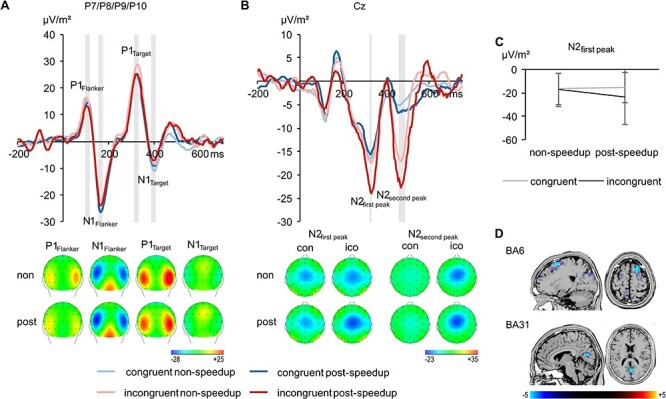
The S-cluster data (*A*) pooled across electrodes P7, P8, P9, and P10 with topographic plots for the incongruent condition separate for nonspeedup (non) and postspeedup (post) trials for each time window at the bottom and (*B*) at electrode Cz with topographic plots for nonspeedup (non) and postspeedup (post) trials in the congruent (con) and incongruent (ico) condition at the bottom. The congruent condition is shown in blue, the incongruent condition in red. The nonspeedup condition is shown in lighter coloring than the postspeedup condition. Time point 0 denotes the time point of the flanker stimulus onset. Time windows used for analyses are highlighted in gray. Topographic plots show the distribution of the potentials at the peak of the respective component. Positive potentials are shown in red, negative potentials are shown in blue, scaling is given in μV/m^2^. The graph in (*C*) shows the interaction of Congruency*PostSpeedup on the first peak of the N2 complex. The sLORETA plots in (*D*) show BA6 as well as BA31 being differentially modulated at the first peak in the N2 time window in the incongruent condition (postspeedup > nonspeedup). For the sLORETA plots, heightened activation is shown in yellow, reduced activation is shown in blue, scaling corresponds to *t*-values.

The RIDE S-cluster data pooled across electrodes P7, P8, P9, and P10 are shown in Figure 3*A*.

For the S-cluster, the repeated-measures ANOVA revealed no significant main effects or interactions on RIDE-P1_Flanker_ (*F* ≤ 3.56; *P* ≥ 0.071) and RIDE-N1_Flanker_ (*F* ≤ 3.21; *P* ≥ 0.085). Regarding the interaction of Congruency*Post-Speedup values of *p*(*H*_0_|*D*) = 0.829 and *p*(*H*_0_|*D*) = 0.701 were obtained, respectively. This indicates positive evidence for the null hypothesis being true for the RIDE-P1_Flanker_ and weak evidence for the null hypothesis being true for the RIDE-N1_Flanker_, underpinning the ANOVA results. The repeated-measures ANOVA on the RIDE-P1_Target_ revealed a main effect of the factor “Electrode” (*F*_2.0,51.6_ = 6.45, *P* = 0.003, η*_p_*^2^ = 0.199). Other main effects and interactions did not reach significance (*F* ≤ 3.89; *P* ≥ 0.059). However, regarding the interaction of Congruency*Post-Speedup (*F*_1,26_ = 3.89, *P* = 0.059, η*_p_*^2^ = 0.130), a value of *p*(*H*_0_|*D*) = 0.031 was obtained, indicating that the alternative hypothesis is likely to be true, contradicting the ANOVA results. Also on the RIDE-N1_Target_, the repeated-measures ANOVA did not reveal any main effects or interactions (*F* ≤ 2.71; *P* ≥ 0.051). Regarding the interaction of Congruency*Post-Speedup, a value of *p*(*H*_0_|*D*) = 0.896 was obtained, indicating positive evidence for the null hypothesis being true, corroborating the ANOVA results.

Concerning the RIDE-N2 complex in the S-cluster, Figure 3*B* shows a “double peak” in the N2 time window on electrode Cz. Both peaks were analyzed separately. A repeated-measures ANOVA on the RIDE-N2_first peak_ revealed a main effect of the factor Congruency (*F*_1,26_ = 6.61, *P* = 0.016, η*_p_*^2^ = 0.203), with larger amplitudes in the incongruent (−20.4 ± 17.5 μV/m^2^) than in the congruent condition (−16.0 ± 12.7 μV/m^2^). Furthermore, analysis revealed an interaction of Congruency*Post-Speedup (*F*_1,26_ = 5.62, *P* = 0.025, η*_p_*^2^ = 0.178; Fig. 3*C*). The speedup-effect was larger in the incongruent (−6.1 ± 16.1 μV/m^2^) than in the congruent condition (1.0 ± 6.7 μV/m^2^; *t*(26) = 2.37, *P* = 0.025). Regarding the interaction of Congruency*Post-Speedup, a value of *p*(*H*_0_|*D*) = 0.003 was obtained, corroborating that the alternative hypothesis is likely to be true. The sLORETA analysis shows that the speedup-associated difference in the incongruent trials on the N2_first peak_ was related to activity modulations in BA31 (posterior cingulate cortex, PCC) and in BA6 (superior frontal gyrus, SFG), with more activation on postspeedup than in nonspeedup trials (Fig. 3*D*). For the RIDE-N2_second peak_, the repeated-measures ANOVA revealed a main effect of the factor Congruency (*F*_1,26_ = 20.89, *P* < 0.001, η*_p_*^2^ = 0.446), with larger amplitudes in the incongruent (−17.5 ± 12.9 μV/m^2^) than in the congruent condition (−5.9 ± 8.4 μV/m^2^). Other main effects or interactions for the N2 peaks did not reach significance (*F* ≤ 1.94, *P* ≥ 0.176). Regarding the interaction of Congruency*Post-Speedup, a value of *p*(*H*_0_|*D*) = 0.876 was obtained, indicating positive evidence for the null hypothesis being true, which underpins the ANOVA results.

**Figure 4:**
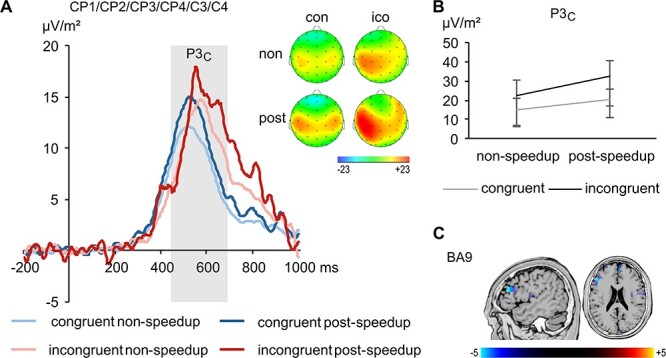
The C-cluster data (*A*) pooled across electrodes CP1, CP2, CP3, CP4, C3, and C4 with topographic plots for nonspeedup (non) and postspeedup (post) trials in the congruent (con) and incongruent (ico) condition. The congruent condition is shown in blue, the incongruent condition in red. The nonspeedup condition is shown in lighter coloring than the postspeedup condition. Time point 0 denotes the time point of the flanker stimulus onset. Time windows used for analyses are highlighted in gray. Topographic plots show the distribution of the potentials at the peak of the respective component. Positive potentials are shown in red, negative potentials are shown in blue, scaling is given in μV/m^2^. The graph in (*B*) shows the interaction of Congruency*PostSpeedup on P3C. The sLORETA plots in (*C*) show BA9 being differentially modulated in the P3C time window in the incongruent condition (postspeedup > nonspeedup). For the sLORETA plots, heightened activation is shown in yellow, reduced activation is shown in blue, scaling corresponds to *t*-values.

The RIDE C-cluster data are shown in Figure 4*A*. A repeated-measures ANOVA on the P3_C_ amplitudes revealed main effects of the factors Congruency (*F*_1,26_ = 55.65, *P* < 0.001, η*_p_*^2^ = 0.682) and Post-Speedup (*F*_1,26_ = 18.82, *P* < 0.001, η*_p_*^2^ = 0.420). Amplitudes were larger in the incongruent (27.2 ± 9.9 μV/m^2^) than in the congruent condition (17.6 ± 7.0 μV/m^2^) and larger in postspeedup (26.2 ± 11.1 μV/m^2^) than in nonspeedup trials (18.6 ± 6.6 μV/m^2^). Furthermore, analysis revealed an interaction of Congruency*Post-Speedup (*F*_1,26_ = 4.51, *P* = 0.043, η*_p_*^2^ = 0.148; Fig. 4*B*). The speedup-effect was larger in the incongruent (10.1 ± 14.3 μV/m^2^) than in the congruent condition (5.1 ± 6.2 μV/m^2^; *t*(26) = −2.12, *P* = 0.043). Regarding the interaction of Congruency*Post-Speedup, a value of *p*(*H*_0_|*D*) = 0.013 was obtained, corroborating that the alternative hypothesis is likely to be true. The sLORETA analysis shows that the speedup-associated difference in the incongruent trials on the P3_C_ was related to activity modulations in the left BA9 (middle frontal gyrus, MFG) with more activation on postspeedup than in nonspeedup trials (Fig. 4*C*). Other main effects or interactions did not reach significance (*F* ≤ 1.85, *P* ≥ 0.140).

**Figure 5:**
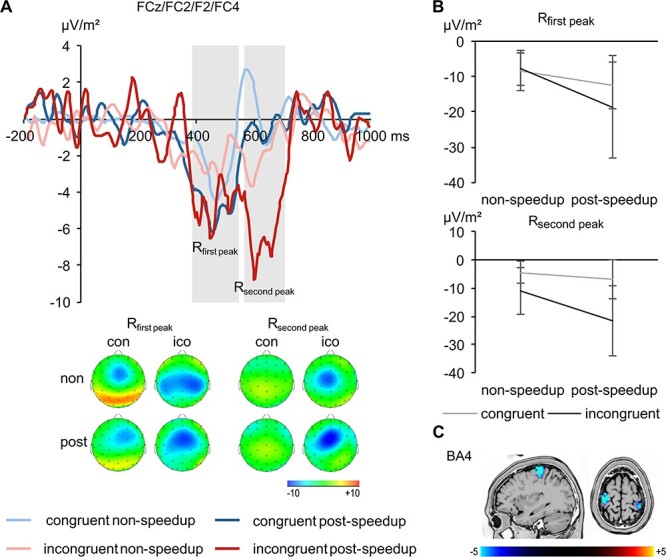
The R-cluster data (*A*) pooled across electrodes FCz, FC2, F2, and FC4 with topographic plots for nonspeedup (non) and postspeedup (post) trials in the congruent (con) and incongruent (ico) condition separate for the first and the second peak at the bottom. The congruent condition is shown in blue, the incongruent condition in red. The nonspeedup condition is shown in lighter coloring than the postspeedup condition. Time point 0 denotes the time point of the flanker stimulus onset. Time windows used for analyses are highlighted in gray. Topographic plots show the distribution of the potentials at the peak of the respective component. Positive potentials are shown in red, negative potentials are shown in blue, scaling is given in μV/m^2^. The graphs in (*B*) shows the interaction of Congruency*PostSpeedup separate for both peaks. The sLORETA plots in (*C*) show BA4 being differentially modulated in the time window of the peaks in the incongruent condition (postspeedup > nonspeedup). For the sLORETA plots, heightened activation is shown in yellow, reduced activation is shown in blue, scaling corresponds to *t*-values.

Concerning the R-cluster, Figure 5*A* shows a double peak in the P3 time window, although the activation does not resemble a classical P3.

Both peaks were analyzed separately. A repeated-measures ANOVA of the R_first peak_ revealed a main effect of the factor Post-Speedup (*F*_1,26_ = 18.57, *P* < 0.001, η*_p_*^2^ = 0.417), with amplitudes being more negative in postspeedup (−15.6 ± 9.4 μV/m^2^) than in nonspeedup trials (−8.1 ± 4.2 μV/m^2^). Also, the main effect of the factor Electrode (*F*_2.4,63.4_ = 10.13, *P* < 0.001, η*_p_*^2^ = 0.280) was significant. Amplitudes were significantly larger at electrodes FCz (−15.0 ± 8.0 μV/m^2^) and FC2 (−13.6 ± 7.8 μV/m^2^) than at electrodes F2 (−10.4 ± 7.1 μV/m^2^) and FC4 (−8.4 ± 4.9 μV/m^2^; |*t*| ≥ 2.17, *P* ≤ 0.39). Furthermore, analysis revealed an interaction of Congruency*Post-Speedup (*F*_1,26_ = 10.28, *P* = 0.004, η*_p_*^2^ = 0.283; Fig. 5*B*). The speedup-effect was larger in the incongruent (−10.8 ± 13.1 μV/m^2^) than in the congruent condition (−4.3 ± 7.0 μV/m^2^; *t*(26) = 3.21, *P* = 0.004). Regarding the interaction of Congruency*Post-Speedup, a value of *p*(*H*_0_|*D*) < 0.001 was obtained, corroborating that the alternative hypothesis is likely to be true. Other main effects or interactions did not reach significance (*F* ≤ 3.04, *P* ≥ 0.093).

For the R_second peak_, a repeated-measures ANOVA revealed main effects of the factors Congruency (*F*_1,26_ = 55.59, *P* < 0.001, η*_p_*^2^ = 0.681) and Post-Speedup (*F*_1,26_ = 16.98, *P* < 0.001, η*_p_*^2^ = 0.395). Amplitudes were more negative in the incongruent (−16.3 ± 7.4 μV/m^2^) than in the congruent condition (−5.6 ± 4.8 μV/m^2^), and more negative in postspeedup (−14.2 ± 7.5 μV/m^2^) than in nonspeedup trials (−7.7 ± 5.2 μV/m^2^). Also, the main effect of the factor Electrode (*F*_2.1,55.3_ = 4.98, *P* = 0.009, η*_p_*^2^ = 0.161) was significant, with larger amplitudes at electrode FCz (−13.8 ± 6.5 μV/m^2^) compared with the others (−10.0 ± 5.1 μV/m^2^; |*t*| ≥ 2.17, *P* ≤ 0.39). Furthermore, analysis revealed an interaction of

“Congruency*Electrode” (*F*_2.2,56.1_ = 8.60, *P* < 0.001, η*_p_*^2^ = 0.249). Flanker congruency effects differed significantly between electrodes FCz (16.1 ± 12.5 μV/m^2^) and F2 (8.1 ± 10.5 μV/m^2^; *t*(26) = 3.36, *P* = 0.002), electrodes FCz and FC4 (5.8 ± 8.2 μV/m^2^; *t*(26) = 3.69, *P* = 0.001), and electrodes FC2 (12.7 ± 9.5 μV/m^2^) and FC4 (*t*(26) = 3.02, *P* = 0.006). Also, the interaction of Congruency*Post-Speedup (*F*_1,26_ = 8.56, *P* = 0.007, η*_p_*^2^ = 0.248; Fig. 5*B*) was significant. The speedup-effect was larger in the incongruent (−10.6 ± 14.5 μV/m^2^) than in the congruent condition (−2.3 ± 5.4 μV/m^2^; *t*(26) = 2.93, *P* = 0.007). Regarding the interaction of Congruency*Post-Speedup, a value of *p*(*H*_0_|*D*) < 0.001 was obtained, corroborating that the alternative hypothesis is likely to be true. Other main effects or interactions did not reach significance (*F* ≤ 2.89, *P* ≥ 0.069). The sLORETA analysis shows that the speedup-elicited difference in the incongruent trials on the double peak time window was related to bilateral activity modulations in BA4 (primary motor cortex), with more activation in postspeedup than in nonspeedup trials (Fig. 5*C*).

## Discussion

Trial-based feedback of response speed is a commonly used part of experimental procedures in areas of cognitive neuroscience dealing with the evaluation of action control processes in clinical and nonclinical populations (Mordkoff and Grosjean 2001; Willemssen et al. 2009; Beste et al. 2010; Perri et al. 2014; van Vliet et al. 2014; Riesel et al. 2019). However, the exact neurophysiological processes modulated by trial-based response time feedback as well as the functional neuroanatomical correlates are largely elusive. To close this gap in knowledge, we examined the effects of trial-based reaction–time feedback in a flanker task using behavioral data and RIDE-decomposed EEG data.

On a behavioral level, general task performance was impaired in the incongruent condition with accuracy rates just above chance level, which might be due to the time pressure induced by the reaction–time feedback. This matches previous findings showing that time pressure reduces performance by impairing the encoding and filtering of stimuli (Dambacher and Hübner 2015). However, immediately after reaction–time feedback was given, the performance improved in the incongruent condition, but was impaired in the congruent condition. These findings can be explained by a narrowing of the attentional focus after reaction–time feedback (Hübner et al. 2010). For the incongruent trials, this narrowing leads to an improved performance because the flanker stimuli are processed less and therefore the conflict between flanker and target stimuli is reduced (Hübner et al. 2010). For the congruent trials, the supporting information of the flanker stimuli is reduced and therefore the performance is impaired (Hübner et al. 2010). The statistical effect sizes show that the modulation was larger for the incongruent trials, indicating that the narrowing of the attentional focus was especially beneficial in the conflicting condition. The interpretation in terms of a narrowing of the attentional focus is also supported by results of the DSTP model (Hübner et al. 2010; Grange 2016), which shows that after the presentation of a speedup signal, the attentional weight, that is, the attentional focus, of the target is heightened compared with the nonspeedup condition (please refer to Supplementary Material S1). However, only the neurophysiological data can provide insights into the processes being modulated.

On a neurophysiological level, a double peak in the N2 time window in the RIDE S-cluster was evident. The second peak of this complex was modulated only by congruency of the flanker information, suggesting that this reflects modulations of the degree of conflict (Folstein and Van Petten 2008). Importantly, the first peak of the N2 complex reflected the interaction found in the behavioral data showing the largest amplitudes when conflict was evident and when reaction–time feedback indicated to respond faster. Particularly, this first peak of a “double-peak N2” can be associated with perceptual inhibition of the processing of irrelevant stimuli (Friedrich and Beste 2018). This is likely to also be the case in the current study, since the inhibition of distractors is crucial in incongruent trials (Stürmer et al. 2000; Verleger et al. 2009; Ocklenburg et al. 2011; Chmielewski et al. 2014; Klein et al. 2014). This process is likely to be enhanced by reaction–time feedback in the previous trial, that is, the perceptual inhibition of irrelevant stimuli is strengthened to achieve a better performance. Furthermore, perceptual inhibition and modulations in the N2 time window are associated with the narrowing of the attentional focus, that is, adapting attentional resources to more demanding tasks (Diamond 2013; Moher et al. 2014; Sänger et al. 2014; Qi et al. 2018), thereby matching the interpretation of the behavioral data. The modulations in the N2 time window were related to a differential activation under conflict in the PCC and SFG, including premotor and supplementary motor areas with greater activation after reaction–time feedback was given. It has been suggested that the PCC is a functional hub for several attention and cognitive control networks (Vincent et al. 2006; Margulies et al. 2009; Leech et al. 2011). Therefore, it is possible that the PCC modulation observed in the current study is associated with the dynamic modulation of the width of the attentional focus and the adjustment of behavioral strategies after trial-based feedback (Hayden et al. 2008; Pearson et al. 2009; Leech and Sharp 2014). For the SFG with its motor-associated areas, previous studies have already suggested that these areas are not only involved in response selection and motor response preparation, but are also relevant for stimulus-related processes. Since the SFG is structurally connected to visual association areas (Hagmann et al. 2008), it is likely to be involved in sensory input integration and to serve as an interface between external stimuli and response selection processes (Mückschel et al. 2017b; Tosun et al. 2017), for example, by adjusting behavioral strategies and inhibiting planned responses in response to cues and conflict detection (Nachev et al. 2007, 2008; Hikosaka and Isoda 2010; Hsu et al. 2011). These adjustments are needed in incongruent trials and are further enhanced after reaction–time feedback, serving as a cue for additional adaptations, which is demonstrated by heightened activation in the current study. Importantly, in the RIDE S-cluster, the proposed modulation of the P1 component was not evident. However, this could not be corroborated by Bayesian statistics, so the effect on the P1 remains inconclusive.

**Figure 6:**
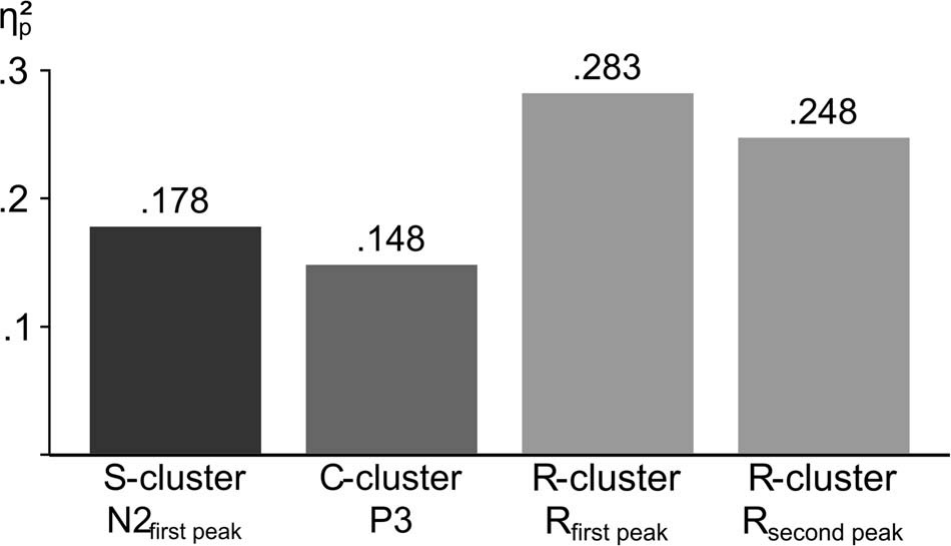
Effect sizes (η*_p_*^2^) for the neurophysiological data in the S-, C-, and R-clusters. Only effect sizes from significant interactions of Congruency* PostSpeedup in the neurophysiological data are displayed.

Crucially, the neurophysiological data suggest that not only attention-related processes, but also response selection processes reflected in the RIDE C-cluster are modulated by trial-based reaction–time feedback. Here, an interaction of incongruent flanker information and reaction–time feedback was evident in the P3 time window, although it was minor compared with the effects seen in the S-cluster according to statistical effect sizes (Fig. 6). However, this modulation in the C-cluster is thought to reflect response selection processes (Mückschel et al. 2014; Verleger et al. 2014), including the refraining from an anticipated response (Takacs et al. 2020) and the updating of task sets (Mückschel et al. 2017a; Wolff et al. 2017). Accordingly, reaction–time feedback might serve as a refreshing cue to recall the instructions, which in the context of a flanker paradigm means to only attend to the central stimulus when selecting the response. For the current study, the data suggest that these processes are associated with the left MFG, a brain region associated with response selection under conflict and high attentional demands (Hadland et al. 2001; Schreiter et al. 2018), as is the case in the incongruent condition after reaction–time feedback. Another interpretation is related to the assumption of a hierarchical organization of the brain relying on the differentiation of forward and backward connections, which reciprocally link different cortical anatomical and functional levels (Friston 2003, 2005). This interpretation is that the P3 in the C-cluster indexes high-order context updating and its amplitude depends on the number of task sets held in prefrontal cortices (Brydges et al. 2020).

Interestingly, considering statistical effect sizes, the modulation by congruency of the flanker information and reaction–time feedback was largest in the P3 time window in the RIDE R-cluster (Fig. 6). However, the observed activation does not resemble a “classical” P3 (Verleger et al. 2005; Twomey et al. 2015), but might represent the more response-related conflict SP. The conflict SP can be associated with conflict processing, especially surrounding the response for appropriate response selection, monitoring, and adaptation processes (West et al. 2005; Chen et al. 2011). In the current study, these processes related to heightened cognitive control are evident under conflict after reaction–time feedback, indicating that this condition requires considerable conflict adaptation. Also, the stronger motor cortex activation observed in the postspeedup trials in the current study might indicate less automatized and more controlled response preparation (Koenraadt et al. 2013). Such a heightened control over processes likely cause time costs, as can be seen in the congruent condition where RTs slow down after the speedup signal. However, in the conflicting condition, these RT costs are not evident, despite a more controlled response preparation according to the neurophysiological data. This might be due to a more efficient and therefore shorter encoding phase because of a sharper attentional focus (Tünnermann et al. 2015), as can be seen in the S-cluster. Indeed, this more controlled response preparation after the narrowing of the attentional focus causes more accurate responses in the conflicting condition. As can be seen, this cascade as proposed by our data emphasizes the role of motor response execution processes, whereas cognitive processes such as modulation of the attentional focus and response selection play a minor part.

Examining this cascade, the current study integrated previous assumptions concerning the effects of response speed strategies in cognitive tasks. On the one hand, it was assumed that focus on response speed mainly affects cognitive processes: Prior hypotheses about modulations often only took the encoding of information, the integration of this information, and the decision process into account (Heitz and Schall 2012; Standage et al. 2014). On the other hand, effects of response strategies including the focus on response speed were analyzed, focusing on motor response processes such as lateralized readiness potentials (Rinkenauer et al. 2004). However, as done in the current study, only the analyses of all processing stages from stimulus encoding up to response motor execution can provide insights in the differential modulations on certain stages and, importantly, allow for a comparison of the modulations of different processes after trial-based reaction–time feedback. Only this comparison could reveal that indeed all the previously proposed processes are modulated by reaction–time feedback, at the same time showing, however, that it mainly influenced motor execution processes.

A limitation of the current study that should be mentioned is the small trial number, especially in the incongruent postspeedup condition. However, data quality is not only ensured by a high number of trials, but also by a reduction of noise in the data (Luck 2014). To decrease the noise in our data, we conducted RIDE analysis, which aligns the residuals of trials and therefore reduces sources of noise in the data, for example, noise arising from smearing due to temporal variability of ERPs (Ouyang et al. 2011, 2015a; Ouyang and Zhou 2020). This reduction of noise was apparently successful, as the analyses yielded reasonable effect sizes, which could not have been generated by noise alone. Furthermore, the achieved effect sizes are higher than the reliably detectable effect sizes according to the sensitivity analysis. Nevertheless, in future studies, a sufficient number of trials also in studies on the effects of reaction–time feedback should be ensured. This could be achieved, for example, through an individually set time limit for each participant according to his RTs, leading to a similar and sufficient number of postspeedup trials for each participant.

In summary, the findings of the current study indicate that reaction–time feedback mainly modulates motor response preparation processes and, to a smaller extent, the width of the attentional focus and response selection processes. This expands previous findings analyzing either cognitive processing aspects such as encoding, information integration, and decision-making when focusing on response speed (Heitz and Schall 2012; Standage et al. 2014) or motor response execution processes (Rinkenauer et al. 2004), since the current study examines all of these processes and allows for comparisons between the processing stages after reaction–time feedback. These comparisons revealed the strongest modulations by focusing on response speed in motor response processes. However, the widely used instruction to respond as quickly as possible to induce this focus on response speed usually aims to modulate cognitive processes from the encoding up to the response selection stage (Heitz and Schall 2012; Standage et al. 2014; Dambacher and Hübner 2015), so that an influence at later stages is not intended. Furthermore, taking into account that the reaction–time feedback interacted with the congruency of the following trial, it is conceivable that the effects of a speed-focused instruction are similar for other conflict-assessing tasks such as the Stroop task (Stroop 1935) or the Simon task (Simon and Small Jr 1969) and therefore induce unintended modulations of conflict effects. This may be subject to future research. Considering these discrepancies between intended and actual effects, the use of instructions concerning response speed should be examined carefully for each research questions addressing processes associated by the brain regions and EEG correlates shown to be modulated by speedup instructions in the current study. The data show how cognitive–neurophysiological are affected when experimenters, sometimes lightheartedly, use task instructions or feedback on participant’s performance.

## Funding

Deutsche Forschungsgemeinschaft (DFG) (SFB 940).

## Notes

The authors thank all participants.

## Availability of Data and Materials

All data as well as summary Statistical Package for the Social Sciences (SPSS) data set used for statistical analysis have been deposed at https://osf.io/72nkh/.

## Supplementary Material

Supplemental_Material_tgab027
