# Supplementary material for: Pushing to the Limits: What Processes during Cognitive Control are Enhanced by Reaction–Time Feedback?
Source: Cereb Cortex Commun. 2021 Apr 7;2(2):tgab027. doi: 10.1093/texcom/tgab027 (PMC8153012; doi:10.1093/texcom/tgab027)
Supplement: Supplemental_Material_tgab027 [file supplemental_material_tgab027.docx]

**Supplementary Material**

**Pushing to the limits: What processes during cognitive control are enhanced by reaction-time feedback?**

Astrid Prochnow, Moritz Mückschel, Christian Beste

**Supplemental Figure 1**


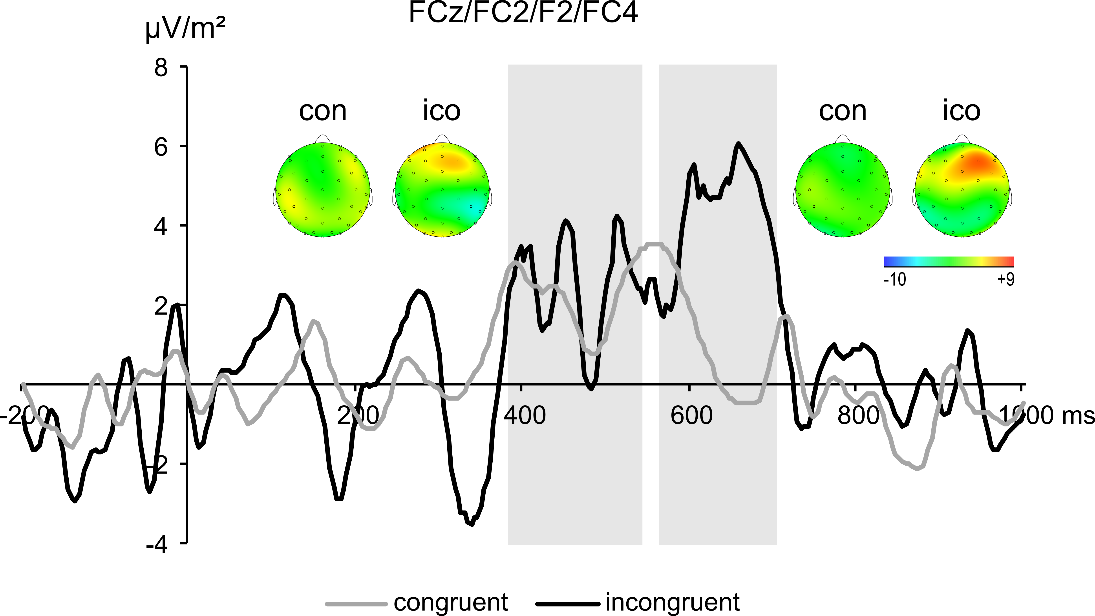


**Supplemental Figure 1.** Difference waves of the R-cluster data at electrodes FCz, FC2, F2 and FC4. Time point zero denotes the time point of the Flanker stimulus onset. Time windows used for analyses are highlighted in grey. Topographic plots show the distribution of the potentials at the peak in the respective time window, i.e. topographic plots on the left side refer to the earlier time window, while topographic plots on the right side refer to the later time window. Positive potentials are shown in red, negative potentials are shown in blue, scaling is given in µV/m².

**Dual-Stage Two-Processes Model**

We analysed the behavioural data with a diffusion model approach to gain further insights into the underlying processes.

*Theoretical background*

We used the Dual-Stage Two-Processes (DSTP) model proposed by Hübner et al. (2010), which divides the processing in a flanker task into two stages of stimulus selection and two phases of response selection. Diffusion drift modelling is applied for the late stage of stimulus selection with boundaries set to C/-D (i.e., flanker and target) and for both phases of response selection with boundaries set to A/-B (i.e., correct and false response). Drift rates represent the increase of accumulated evidence per second. The early stage of stimulus selection is associated with sensory filtering and setting the attentional weights, i.e. the attentional focus, of target and flanker stimuli (i.e., drift rates for target µ_ta_ and flanker µ_fl_). The settings from this stage sum up to the drift rate in the first phase of response selection, µ_RS1_. In parallel with the first phase of response selection, the late stage of stimulus selection runs with the drift rate µ_SS_. If the diffusion process in the first phase of response selection hits the boundary A/-B first, the corresponding response is selected and executed. If the diffusion process in the late stage of stimulus selection hits the boundary C/-D first, the second phase of response selection is entered at the evidence level of the first phase of response selection to that time point and the drift rate µ_RS2_ depending on the selected stimulus. If the diffusion process in the second phase of response selection hits the boundary A/-B, the corresponding response is selected and executed. For more details, please refer to Hübner et al. (2010) and Dambacher and Hübner (2015).

*Methods*

Analyses were conducted with the R-package *flankr* provided by Grange (2016), which fits congruent and incongruent data simultaneously and provides values for response selection boundary A, stimulus selection boundary C, drift rates µ_ta_, µ_fl_, µ_SS_ and µ_RS2_, as well as the non-decision time T_er_. We fitted multiple DSTP models to the data with 40 random starting parameters and a variance of 10 for 100000 trials separate for non-speedup and post-speedup trials. The script used to fit the DSTP model to the human data is deposed under [URL]. For the fitting procedure data were presented in the form of cumulative distribution functions (CDFs) for correct responses and conditional accuracy functions (CAFs) to account for accuracy performance, dividing the data into bins. The degree of fit between the modelled data and the human data was assessed by the likelihood ratio chi-square statistics, which is calculated as follows:

$G^{2}=2\sum_{i}^{J} N p_{i}\ln\left( \frac{p_{i}}{\pi_{i}} \right)$,

with *p_i_* as the proportion of human observations in the *i*th bin, *π_i_* as the proportion in this bin predicted by the model, *N* as the average number of trials and *J* as the total number of bins. To allow for statistical comparisons, model fittings were conducted on the single subject level, resulting in fitting parameters for each participant for both reaction-time feedback conditions. Conditions were then compared using paired t-tests.

*Results*

Model fit for the non-speedup condition was significantly better than model fit for the post-speedup condition (G²_non_ = 91.23 ± 37.56; G²_post_ = 185.01 ± 75.51; t(26) = -6.19, p < .001).

The mean and standard deviation of the mean of the fitting parameters separate for the reaction-time conditions are displayed in Tab. S1, the results of the t-tests between conditions are displayed in Tab. S2.

*Table S1*

*Parameter estimates from DSTP model fits (mean, standard deviation of the mean) separate for the reaction-time conditions*

|  | A/-B | C/-D | µ_ta_ | µ_fl_ | µ_RS1_ | µ_SS_ | µ_RS2_ | T_er_ |
| --- | --- | --- | --- | --- | --- | --- | --- | --- |
| non-speedup | .051  ± .010 | .137  ± .032 | .038  ± .035 | .237  ± .079 | .276  ± .076 | .800  ± .148 | 1.000  ± .244 | .131  ± .038 |
| post-speedup | .055  ± .012 | .124  ± .038 | .069  ± .066 | .205  ± .081 | .275  ± .110 | .716  ± .170 | 1.135  ± .273 | .134  ± .042 |

*Notes.* A/-B response selection boundaries, C/-D stimulus selection boundaries, µ_ta_ drift rates for the target in phase 1, µ_fl_ drift rates for the flanker in phase 1, µ_RS1_ drift rates for the response selection in phase 1 (calculated as µ_ta_ + µ_fl_), µ_SS_ drift rates for the late stage of stimulus selection, µ_RS2_ drift rates for the response selection in phase 2, T_er_ non-decision time (in seconds).

Table S2

*Results of paired t-tests between the reaction-time conditions*

| Parameter | *t* | *df* | *p* |
| --- | --- | --- | --- |
| A/-B | -1.45 | 26 | .161 |
| C/-D | 1.54 | 26 | .135 |
| µ_ta_ | -2.24 | 26 | .034 |
| µ_fl_ | 1.65 | 26 | .111 |
| µ_RS1_ | .06 | 26 | .955 |
| µ_SS_ | 2.16 | 26 | .040 |
| µ_RS2_ | -1.94 | 26 | .063 |
| T_er_ | -.44 | 26 | .664 |

*Notes.* p-values are uncorrected.

*Discussion*

According to the Dual-Stage Two-Processes (DSTP) model proposed by Hübner et al. (2010), the parameters in this model refer to specific cognitive processes. Results of the fitting procedure of our data to this model revealed a significant increase of the drift rate of the target in the post-speedup condition, but this did not change the summed up drift rate of the first phase of response selection RS1. Further, the drift rate of the late stage of stimulus selection was decreased in the post-speedup condition, prolonging the time until this process hits the corresponding boundary C/-D. Taking the competition of the late stage of stimulus selection and the first phase of response selection into account, this leads to an increased probability of the first phase of response selection hitting the boundary A/-B first. Therefore, in the post-speedup condition, it is more likely that response selection is based on the early and less sophisticated processes. However, since the weight of the target stimulus within the drift rate RS1, i.e. the attentional focus on the target, is higher in the post-speedup condition, this leads to a better performance in the incongruent condition, because the processing of the target stimulus is intensified, whereas the focussing on the target leads to a slightly worse performance in the congruent condition.

To explain these opposed effects of the speedup signal on the congruent and the incongruent condition, it would be important to analyse also these conditions separately and compare them. Further, for future studies including model fitting, it would be important to take care of a sufficient trial number in each condition.
